# Population Structure of the Malaria Vector *Anopheles sinensis* (Diptera: Culicidae) in China: Two Gene Pools Inferred by Microsatellites

**DOI:** 10.1371/journal.pone.0022219

**Published:** 2011-07-22

**Authors:** Yajun Ma, Manni Yang, Yong Fan, Jing Wu, Ying Ma, Jiannong Xu

**Affiliations:** 1 Department of Pathogen Biology, Second Military Medical University, Shanghai, China; 2 Department of Biology, New Mexico State University, Las Cruces, New Mexico, United States of America; Global Viral Forecasting Initiative, United States of America

## Abstract

**Background:**

*Anopheles sinensis* is a competent malaria vector in China. An understanding of vector population structure is important to the vector-based malaria control programs. However, there is no adequate data of *A. sinensis* population genetics available yet.

**Methodology/Principal Findings:**

This study used 5 microsatellite loci to estimate population genetic diversity, genetic differentiation and demographic history of *A. sinensis* from 14 representative localities in China. All 5 microsatellite loci were highly polymorphic across populations, with high allelic richness and heterozygosity. Hardy–Weinberg disequilibrium was found in 12 populations associated with heterozygote deficits, which was likely caused by the presence of null allele and the Wahlund effect. Bayesian clustering analysis revealed two gene pools, grouping samples into two population clusters; one includes six and the other includes eight populations. Out of 14 samples, six samples were mixed with individuals from both gene pools, indicating the coexistence of two genetic units in the areas sampled. The overall differentiation between two genetic pools was moderate (*F*_ST_ = 0.156). Pairwise differentiation between populations were lower within clusters (*F*_ST_ = 0.008–0.028 in cluster I and *F*_ST_ = 0.004–0.048 in cluster II) than between clusters (*F*_ST_ = 0.120–0.201). A reduced gene flow (*Nm = *1–1.7) was detected between clusters. No evidence of isolation by distance was detected among populations neither within nor between the two clusters. There are differences in effective population size (*Ne* = 14.3-infinite) across sampled populations.

**Conclusions/Significance:**

Two genetic pools with moderate genetic differentiation were identified in the *A. sinensis* populations in China. The population divergence was not correlated with geographic distance or barrier in the range. Variable effective population size and other demographic effects of historical population perturbations could be the factors affecting the population differentiation. The structured populations may limit the migration of genes under pressures/selections, such as insecticides and immune genes against malaria.

## Introduction

*Anopheles sinensis* Wiedemann 1828 is a widely distributed Oriental species [1], [2], [3]. In China, *A. sinensis* was incriminated as a competent malaria vector and was responsible for the transmission during the recurrence of vivax malaria in recent years [4]. Genetically based methods have been proposed for malaria vector control. These methods focus mainly in altering vectorial capacity through the genetic modification of natural vector populations by means of introducing refractoriness genes or by sterile insect technologies [5]. Knowledge of the genetic structure of vector species is, therefore, an essential requirement as it should contribute not only to predict the spread of genes of interest, such as insecticide resistance or refractory genes, but also to identify heterogeneities in disease transmission due to distinct vector populations [6]. A complete understanding of vector population structure and the processes responsible for the distribution of differentiation is important to vector-based malaria control programs and for identifying heterogeneity in disease transmission as a result of discrete vector populations [7]. Susceptibility to *Plasmodium* infection, survival and reproductive rates, degree of anthropophily, and the epidemiology of malaria in the human host may all be affected by genetic variation in vector populations [8].

*A. sinensis* exhibits variation in ecology [9], morphology [9], [10], chromosomes [9], [11], isozymes [9], mtDNA [12], random amplified polymorphic DNA (RAPDs) [13], and rDNA second internal transcribed spacer (ITS2) sequences [14]. Cytogenetic studies have revealed two karyotypic forms, A (XY1) and B (XY2), in *A. sinensis* [11], which have distinct ITS2 sequences [14]. Both forms exist in Thailand [11], and only form B occurs in China and Korea [14], [15]. The susceptibility to malaria varies in different geographic areas. In Thailand, wild *A. sinensis* was poorly susceptible to *Plasmodium vivax* [16], so were the laboratory lines of forms A and B [17]. In China, *A. sinensis* is more susceptible to *P. vivax* than to *P. falciparum,* and therefore it is an important vector in the areas where no other vector species exist [22], [23]. In Korea, *A. sinensis* was incriminated as a competent malaria vector [18], [19] and was responsible for the transmission during the recurrence of vivax malaria in recent years [20], [21]. In Japan, due to its abundance *A. sinensis* has long been suspected to be the most important vector of malaria in temperate Japan, including Okinawa and Hokkaido Islands [2], [3].

Despite its significance in malaria transmission, only a few studies on population genetics have been conducted [12], [13]. Microsatellites are highly polymorphic genetic markers that evolve much faster than mitochondrial or nuclear genes, and are particularly useful for resolving the structure of populations at a finer geographical and evolutionary scale. They have been extensively used for population studies of anophelines, such as *A. darlingi* [24], [25], [26], *A. moucheti* [27], *A. gambiae* [28] and *A. nili* [29]. Microsatellite DNA markers have been isolated from *A. sinenesis* [30], [31]. In this study, we have used microsatellite markers to estimate levels of genetic differentiation among populations of *A. sinensis* to determine the population structure across its range in China.

## Results

### Population sampling and species identification

**Figure 1:**
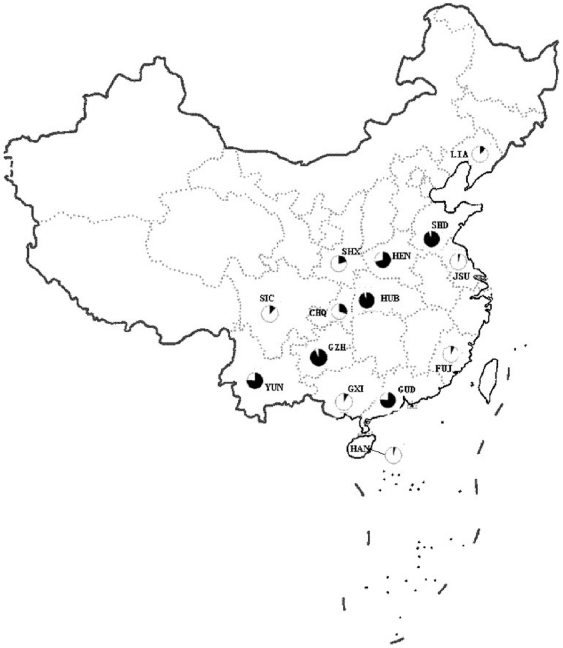
A schematic map of China showing sampling sites for *A. sinensis.* The population genetic affiliation to the two clusters in each locality was displayed by a pie chart with black as cluster I and white as cluster II (see Table 3 for details).

Fourteen samples of wild mosquitoes were collected from 20 locations in China (Figure 1, Table 1). A total of 327 female *A. sinensis* were identified by a species diagnostic PCR assay [32]. Five samples, YUN, HUB, LIA, SHD and SIC, consisted of specimen pooled from two or three collections, as stated in Table 1.

**Table 1:** *Anopheles sinensis* collections in China.

Code	Collection site	Latitude/Longitude Coordinates	Sample size	Date
YUN	Yanjin, YunnanPuer, Yunnan	104°13′ E, 28°06′ N101°11′ E, 23°06′ N	309	7/068/05
HUB	Guangshui, HubeiSuizhou, Hubei	113°47′ E, 31°41′ N113°15′ E, 31°52′ N	237	7/077/07
GZH	Congjiang, Guizhou	108°41′ E, 25°43′ N	27	8/07
HEN	Tongbai, Henan	113°23′ E, 32°29′ N	26	8/07
GUD	Zhuhai, Guangdong	113°30′ E, 22°17′ N	30	10/07
SHX	Ningshan, Shaanxi	108°25′ E, 33°31′ N	418	7/077/97
LIA	Xingchen, LiaoningSuizhong, Liaoning	120°25′ E, 40°33′ N119°58′ E, 40°16′ N	65	7/089/08
SHD	Donge, ShandongYutai, ShangdongWeishan, Shangdong	116°15′ E, 36°18′ N116°33′ E, 34°59′ N116°58′ E, 34°52′ N	11114	7/067/067/06
CHQ	Wanxian, Chongqing	108°22′ E, 31°15′ N	25	8/08
GXI	Tiane, Guangxi	106°59′ E, 25°01′ N	16	7/05
SIC	Pujiang, SichuanPixian, Sichuan	103°29′ E, 30°14′ N103°53′ E, 30°50′ N	128	7/967/97
FUJ	Jiangyang, Fujian	118°02′ E, 27°24′ N	20	9/97
HAN	Chengmai, Hainan	109°58′ E, 19°39′ N	15	7/96
JSU	Wujin, Jiangsu	119°54′ E, 31°44′ N	20	7/97

### Genetic variability within populations

Polymorphism at five microsatellite loci varied, with number of alleles (*A*) as 12 (ANS15), 17 (ANS1 and ANS6), 18 (ANS11) and 19 (ANS5), respectively (Table 2). The average number of alleles per locus was in a range between 6.5 (ANS5) and 10.4 (ANS11). The minimum mean number of alleles of all loci was in LIA population (6.2), and the maximum in YUN (10.2). Allele distributions across populations were depicted in Figure S1. The average observed heterozygosity (*H_O_*) across all samples ranged from 0.398 (ANS5) to 0.757 (ANS11), the minimum *Ho* was in GUD (0.433), the maximum *Ho* in SIC (0.760). To determine if the null alleles impacted the population genetic analyses, we performed these analyses both before and after the dataset were adjusted for estimated null allele frequencies. The effect of this treatment was minimal and did not significantly change the degree or statistical significance of the estimated parameters.

**Table 2:** Summary of microsatellite variation at 5 loci for *A. sinensis* in China.

Locus		HEN	YUN	GUD	SHX	GZH	HUB	SHD	CHQ	LIA	FUJ	GXI	HAN	JSU	SIC	all samples
(n)		N = 26	N = 39	N = 30	N = 22	N = 27	N = 30	N = 26	N = 25	N = 11	N = 20	N = 16	N = 15	N = 20	N = 20	N = 327
ANS1	*A*	10	9	12	10	11	13	10	9	8	9	7	8	11	10	9.786
	*R_S_*	3.328	3.027	2.772	2.69	3.092	3.337	3.207	2.946	3.391	2.856	2.899	3.167	3.383	2.908	3.072
	*H*_E_	0.877	0.812	0.729	0.701	0.818	0.877	0.849	0.786	0.892	0.75	0.777	0.844	0.888	0.767	0.812
	*H*_O_	0.391	0.486	0.500	0.650	0.630	0.586	0.714	0.417	0.273	0.500	0.667	0.733	0.789	0.700	0.574
	*r*	0.251	0.175	0.127	0.020	0.096	0.132	0.063	0.199	0.313	0.134	0.048	0.045	0.040	0.027	0.119
	*F*_IS_	0.560*	0.406*	0.318*	0.075	0.233*	0.335*	0.162	0.475*	0.704*	0.339	0.146	0.135	0.113	0.089	0.151
ANS5	*A*	6	13	8	7	6	5	3	6	3	6	6	8	7	7	6.500
	*R_S_*	2.616	3.143	2.482	2.737	2.345	2.093	1.547	2.498	1.675	2.258	2.062	2.155	2.099	2.918	2.331
	*H*_E_	0.695	0.829	0.654	0.736	0.624	0.520	0.28	0.658	0.329	0.585	0.480	0.510	0.514	0.789	0.586
	*H*_O_	0.333	0.447	0.300	0.476	0.346	0.393	0.273	0.333	0.182	0.400	0.250	0.533	0.550	0.750	0.398
	*r*	0.207	0.181	0.209	0.141	0.165	0.078	0.001	0.189	0.101	0.108	0.147	-	-	0.011	0.110
	*F*_IS_	0.526*	0.464*	0.546*	0.359*	0.451*	0.248	0.027	0.499*	0.459	0.321	0.487	-0.047	-0.072	0.050*	0.203
ANS11	*A*	10	12	11	10	12	12	10	11	6	11	12	8	10	10	10.357
	*R_S_*	3.308	3.434	3.334	3.276	3.387	3.482	3.018	3.295	3.153	3.383	3.282	3.116	3.372	3.152	3.285
	*H*_E_	0.870	0.899	0.875	0.862	0.887	0.907	0.796	0.868	0.844	0.887	0.859	0.825	0.886	0.836	0.864
	*H*_O_	0.640	0.714	0.800	0.857	0.800	0.828	0.783	0.739	0.636	0.850	0.813	0.533	0.750	0.850	0.757
	*r*	0.115	0.091	0.032	-	0.037	0.034	-	0.059	0.094	-	0.011	0.147	0.061	-	0.049
	*F*_IS_	0.269*	0.207*	0.087	0.006	0.100	0.089	0.017	0.151	0.255	0.043	0.056	0.362	0.157	-0.017	0.109
ANS15	*A*	6	7	5	6	7	6	6	6	5	7	5	8	8	10	6.571
	*R_S_*	2.841	2.806	2.356	2.831	2.605	2.855	2.788	2.809	2.535	3.043	2.510	3.344	3.061	3.030	2.815
	*H*_E_	0.766	0.757	0.639	0.762	0.697	0.775	0.759	0.766	0.668	0.817	0.686	0.883	0.819	0.805	0.757
	*H*_O_	0.308	0.500	0.100	0.476	0.444	0.321	0.550	0.067	0.400	0.900	0.688	0.733	0.950	0.950	0.528
	*r*	0.253	0.141	0.325	0.153	0.142	0.250	0.109	0.387	0.144	-	-	0.065	-	-	0.141
	*F*_IS_	0.603*	0.343	0.846*	0.381	0.366*	0.590*	0.281	0.916*	0.415	-0.105	-0.003	0.174	-0.165	-0.186	0.126
ANS6	*A*	7	10	7	9	10	9	9	9	9	8	6	9	12	7	8.643
	*R_S_*	3.063	3.056	2.964	3.28	3.315	3.027	3.251	3.191	3.417	3.224	2.749	3.156	3.222	2.892	3.129
	*H*_E_	0.822	0.808	0.797	0.868	0.874	0.810	0.859	0.848	0.896	0.858	0.735	0.839	0.851	0.774	0.831
	*H*_O_	0.583	0.519	0.467	0.765	0.500	0.522	0.500	0.652	0. 909	0.900	0.429	0.467	0.650	0.550	0.577
	*r*	0.123	0.153	0.178	0.042	0.192	0.151	0.185	0.097	-	-	0.164	0.190	0.049	0.117	0.117
	*F*_IS_	0.295	0.363*	0.419*	0.122	0.433*	0.361	0.423*	0.235	-0.015	-0.051	0.426	0.453*	0.241	0.295	0.212
All loci	*A*	7.800	10.200	8.600	8.400	9.200	9.000	7.600	8.200	6.200	8.200	7.200	8.200	9.600	8.800	8.371
	*R_S_*	3.048	3.093	2.792	2.972	2.949	2.959	2.762	2.948	2.834	2.959	2.700	2.988	3.027	2.980	2.929
	*H*_E_	0.806	0.821	0.739	0.786	0.780	0.778	0.709	0.785	0.726	0.779	0.707	0.780	0.792	0.794	0.770
	*H*_O_	0.451	0.533	0.433	0.645	0.544	0.530	0.564	0.442	0.480	0.701	0.569	0.600	0.738	0.760	0.571
	*r*	0.190	0.148	0.174	0.071	0.127	0.129	0.072	0.186	0.130	0.048	0.074	0.089	0.030	0.031	0.107
	*F*_IS_	0.445	0.354	0.418	0.183	0.307	0.323	0.208	0.444	0.350	0.091	0.201	0.237	0.070	0.044	0.263

First row indicates collection sites and sample size in parenthesis; *A,* number of alleles; *R_S_,* allelic richness; *H*_E_, expected heterozygosity; *H*_O_, observed heterozygosity; *r*, estimated frequency of null alleles; *F*_IS_, inbreeding coefficient; All loci/samples, mean values over loci or populations; -, no significant heterozygote deficiency;

*, Probability test against Hardy- Weinberg proportions (*P*<0.001).

The Hardy-Weinberg exact tests were performed for five loci. No locus was in Hardy-Weinberg equilibrium (HWE) for all the samples assayed. At the population level, 26 out of 70 (37.14%) comparisons did not conform to Hardy-Weinberg expectations after sequential Bonferroni correction, and the deviations were associated with positive inbreeding coefficient (*F*_IS_), reflecting heterozygosity deficits (Table 2). Significant deviation from HWE varied across loci in a population -dependent manner. The YUN, HEN, GUD and GZH populations had the highest number of loci in departure from HWE (4 of 5), while the SHD, SHX, LIA, HAN and SIC populations had the fewest (1 of 5). The FUJ, GXI and JSU populations had no loci in departure from HWE (Table 2). In all samples, some specimen failed to amplify at one locus while succeeded at the remaining loci, suggesting the presence of null alleles. Estimates of the frequency of null alleles are given in Table 2. The locus ANS15, for example, showed both high *F*_IS_ values and high frequencies of null alleles in samples CHQ, GUD, HEN and HUB.

Fisher's exact tests were conducted for linkage disequilibrium (LD) within each of the 14 collections. Out of 140 comparisons only five pairs (3.57%) were at LD (*P*<0.05). Two pairs were detected in HAN (ANS5/ANS15, ANS1/ANS5) and the other three pairs were in SHD (ANS6/ANS15), HUB (ANS1/ANS6), GUD (ANS6/ANS11), respectively (Table 2). No pair of loci appeared in LD in more than one population, suggesting genetic independence between loci. When the test was performed in the pooled populations, no pairs of loci out of 10 possible combinations showed significant *P* values (*P*>0.05).

### Genetic differentiation among populations

**Figure 2:**
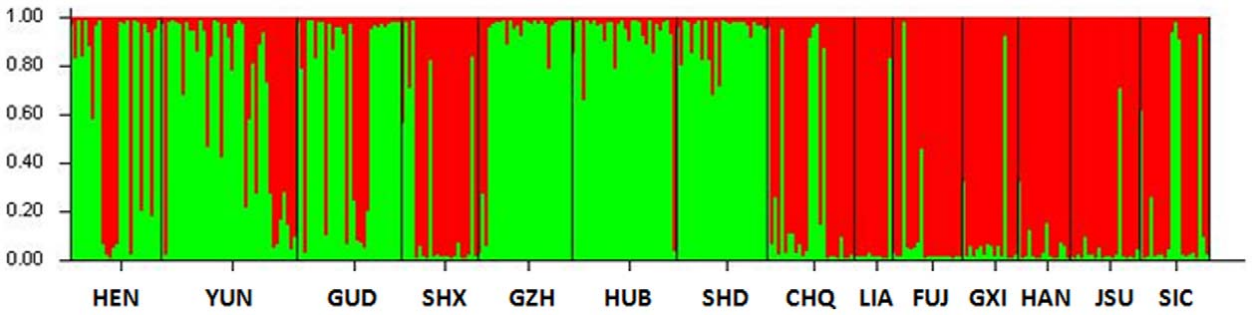
Bayesian cluster analysis using STUCTURE. Graphical representation of the data set for the most likely *K* (*K* = 2), where each color corresponds to a suggested cluster and each individual is represented by a vertical bar. The X-axis corresponds to population codes. The Y-axis presents the probability of assignment of an individual to each cluster.

The significant deviations from HWE with heterozygote deficiency and the presence of LD suggest the presence of population subdivision within samples (the Wahlund effect). We therefore examined if there were different gene pools in these samples. The Bayesian cluster analysis divided populations into two main subgroups (posterior probability of Bayesian clustering Ln(D) likelihood score optimal for k = 2 clusters) (Figure 2). One gene pool (cluster I) was composed of GZH, HUB, SHD, HEN, GUD and YUN, and the other (cluster II) was composed of LIA, CHQ, FUJ, GXI, HAN, JSU, SIC and SHX. Allele composition varied to a limited extent among populations within the clusters but varied considerably between the clusters. For example, 3 alleles at locus ANS1, 4 alleles at ANS5 and 3 alleles at ANS6 were differentially distributed among populations between the clusters (Figure S2). In different localities, there are different proportions of individuals from different gene pools (Table 3). For example, in the HEN 70% of individuals were assigned to the cluster I and the remaining 30% to the cluster II; an opposite occurred in CHQ, in which 69.8% of individuals belong to the cluster II and 30.2% to the cluster I. In total, six samples (three in each cluster) were mixed with at least 15% of individuals assigned to the other cluster (Figure 1 and Table 3), indicating the coexistence of two gene pools in these localities.

**Table 3:** Probability of assignment of individuals to each population cluster.

Cluster	Populations	Probability of assignment of individuals to each cluster		Number of loci in departure from HWE
		I	II	
I	SHD	0.939	0.061	1
	HUB	0.929	0.071	2
	GZH	0.882	0.118	4
	YUN	0.774	0.226	4
	GUD	0.756	0.244	4
	HEN	0.700	0.300	4
II	JSU	0.039	0.961	0
	HAN	0.046	0.954	1
	FUJ	0.073	0.927	0
	GXI	0.082	0.918	0
	LIA	0.121	0.879	2
	SIC	0.166	0.834	1
	SHX	0.213	0.787	1
	CHQ	0.302	0.698	3

Overall genetic divergence between two gene pools was assessed. First, specimens were assigned to the cluster I or II at greater than 80% probabilities, which resulted in 126 individuals in the cluster I and 135 individuals in the cluster II. Then the two pools of individuals were analyzed by the Wright's *F* statistics (Table 4). The overall value of *F*_IT_ (0.379) showed significant heterozygote deficits at the total population level, most likely due to the presence of null alleles. Individually, three loci, ASN1, ASN6 and ASN11, presented significant heterozygote reduction at the population level (*P*<0.05). The average value of *F*_IS_ (0.264) showed heterozygote reduction at the subpopulation level. These results corroborated the homozygote excess detected with the individual HWE tests (Table 2). Locus ANS5 had the highest *F*_ST_ value (0.545) while other loci ranged between 0.020–0.086. The average *F*_ST_  = 0.156, indicating a moderate genetic heterogeneity between the two gene pools. An AMOVA analysis using the two clusters found that 15.6% of the variance was attributed to between populations and 84.4% to within populations.

**Table 4:** Wright's *F* statistics values per locus between two clusters.

Locus	*F*_IT_	*F*_ST_			*F*_IS_
			*P1*	*P2*	
ANS1	0.344; ***P*** ** = 0.041**	0.047	0.259	0.259	0.312; *P* = 0.571
ANS5	0.626; *P* = 0.543	0.545	0.08	0.08	0.177; *P* = 0.373
ANS11	0.156; ***P*** ** = 0.018**	0.020	0.124	0.124	0.138; *P* = 0.772
ANS15	0.402; *P* = 0.072	0.086	0.266	0.266	0.346; *P* = 0.504
ANS6	0.345; ***P*** ** = 0.032**	0.036	0.277	0.277	0.321; *P* = 0.586
Total	0.379; *P = 0.707*	0.156	1.006	1.006	0.264; *P* = 2.807
CI	0.229–0.574	0.026–0.452			0.148–0.335

CI = 99% confidence internal. *P1* = assuming non-random breeding; *P2* = assuming random breeding.

Then we tested genetic heterogeneity among populations within and between the two gene pools. We chose populations in which more than 85% individuals were assigned to the cluster I or II for the analysis. The resultant populations included three (GZH, HUB and SHD) from the cluster I and five (LIA, FUJ, GXI, HAN and JSU) from the cluster II. Table 5 shows *F*_ST_ estimates for pairwise comparisons among populations. Within the clusters, paiwise *F*_ST_ was low, 0.008 (HUB-GZI) to 0.028 (GZH-SHD) in the cluster I, and 0.004 (JSU-LIA) to 0.048 (HAN-GXI) in the cluster II. Between the clusters, higher level of differentiation was demonstrated with *F*_ST_ from 0.120 (FUJ-GZH) to 0.201 (SHD-GXI). Of 28 pairwise comparisons, 17 were significant including all 15 comparisons between clusters (*P*<0.05). *Nm* estimates among populations were higher (4.93 to 65.54) within the clusters, and much lower (0.99 to 1.84) between the clusters (Table 5).

**Table 5:** Pairwise genetic distance (*F*_ST_) and gene flow (*Nm*) for populations of *A. sinensis*.

Cluster Population		I			II				
		GZH	HUB	SHD	LIA	FUJ	GXI	HAN	JSU
I	GZH		30.614	8.616	**1.389**	**1.833**	**1.471**	**1.656**	**1.684**
	HUB	0.008(827)		12.839	**1.377**	**1.726**	**1.373**	**1.475**	**1.656**
	SHD	0.028(1372)	0.019(558)*		**1.088**	**1.292**	**0.993**	**1.083**	**1.193**
II	LIA	**0.153(1966)** *	**0.154(1131)** *	**0.187(585)** *		6.277	5.381	5.537	65.539
	FUJ	**0.120(963)** *	**0.127(629)** *	**0.162(1004)** *	0.038(1451)		6.434	5.564	27.528
	GXI	**0.145(194)** *	**0.154(1007)** *	**0.201(1541)** *	0.044(2126)	0.037(1166)*		4.926	9.256
	HAN	**0.131(720)** *	**0.145(1418)** *	**0.188(1984)** *	0.043(2533)	0.043(1233)	0.048(709)		13.263
	JSU	**0.129(1292)** *	**0.131(572)** *	**0.173(600)** *	0.004(964)	0.009(510)	0.026(1484)	0.019(1703)	

The pairwise *Nm* values are above diagonal; pairwise *F* **_ST_** values below diagonal and within population along the diagonal.

*, *P*<0.05 after sequential Bonferroni correction. The bold values are comparison between clusters. Approximate geographical distances in km are in parentheses. Abbreviations of localities are in Table 1.

**Figure 3:**
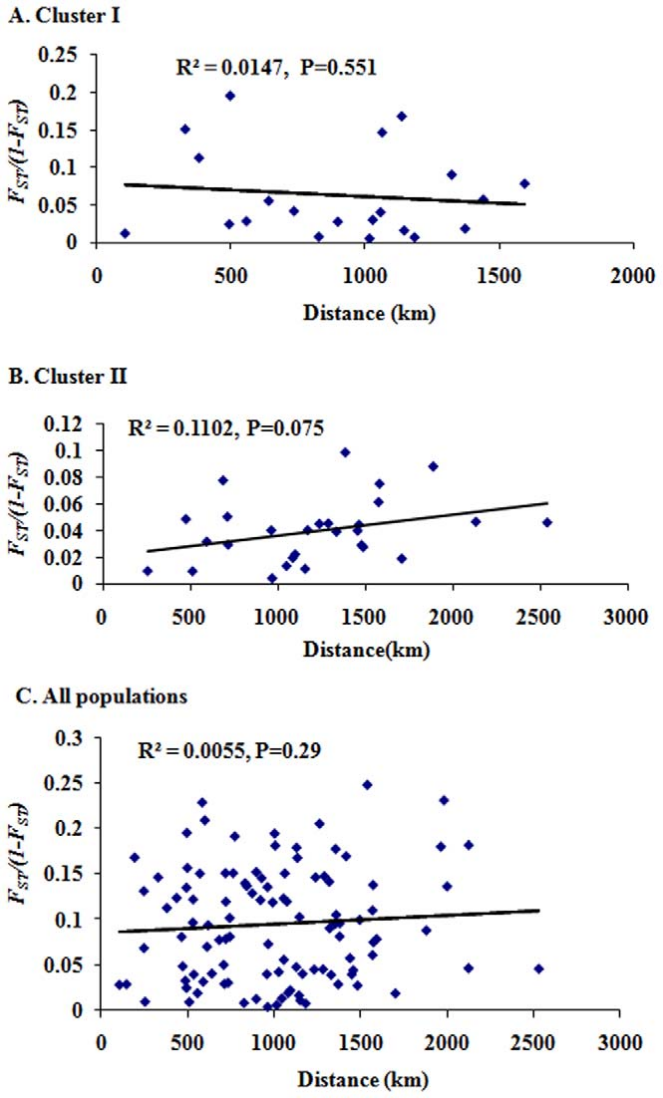
Correlation between average *F*_ST_ estimates and geographic distance between collection sites for pairwise comparisons of *A. sinensis* populations. A, populations of cluster I; B, populations of cluster II; C, all populations.

Tests of isolation by distance were performed for each population cluster and for all of the populations together. No statistically significant correlations were detected between genetic differentiation and geographic distances based on the Mantel test in all cases (Figure 3). The results suggest that geographic distance does not significantly contribute to the genetic differentiation observed in *A. sinensis* populations.

### Effective population size and demographic stability

Estimates of expected heterozygosity under mutation-drift equilibrium (MDE) were calculated for detecting demographic instability. These statistics are expected to be equal in a neutral locus at MDE. Results of the heterozygosity tests (Table 6) did not reveal any evidence for departure from MDE in two models (SMM and TMP) in any of the 14 populations. However, a consistent trend for lower-than-expected heterozygosity (i.e., *He<Heq*) was detected in the GXI and SIC populations under SMM model (*P*<0.05), suggesting a recent demographic expansion.

**Table 6:** P-value for the heterozygosity tests for each *A. sinensis* population.

Cluster	Population		TPM			SMM
			70%^a^	80% a	90% a	
I	HEN	*He>Heq*	4	4	4	4
		*Sign test*	0.333	0.329	0.322	0.324
		*Wilcoxon test*	0.063	0.063	0.063	0.063
	YUN	*He>Heq*	3	3	3	1
		*Sign test*	0.666	0.681	0.67	0.097
		*Wilcoxon test*	0.813	1	1	0.156
	GUD	*He>Heq*	3	3	2	2
		*Sign test*	0.673	0.67	0.34	0.35
		*Wilcoxon test*	0.813	0.813	0.625	0.156
	GZH	*He>Heq*	2	2	2	1
		*Sign test*	0.325	0.33	0.342	0.094
		*Wilcoxon test*	1	0.813	0.156	0.094
	HUB	*He>Heq*	3	2	2	2
		*Sign test*	0.678	0.33	0.328	0.333
		*Wilcoxon test*	0.625	1	1	0.625
	SHD	*He>Heq*	3	3	2	2
		*Sign test*	0.666	0.66	0.329	0.34
		*Wilcoxon test*	1	0.813	0.625	0.156
II	CHQ	*He>Heq*	3	3	3	1
		*Sign test*	0.661	0.677	0.678	0.094
		*Wilcoxon test*	0.625	1	0.813	0.063
	LIA	*He>Heq*	3	3	3	3
		*Sign test*	0.66	0.661	0.659	0.681
		*Wilcoxon test*	0.219	0.813	0.813	1
	FUJ	*He>Heq*	3	3	3	3
		*Sign test*	0.671	0.673	0.663	0.676
		*Wilcoxon test*	0.813	0.813	0.813	0.813
	GXI	*He>Heq*	1	1	1	0
		*Sign test*	0.086	0.094	0.087	0.010*
		*Wilcoxon test*	0.156	0.094	0.063	0.031*
	HAN	*He>Heq*	3	3	2	2
		*Sign test*	0.675	0.68	0.329	0.336
		*Wilcoxon test*	0.813	1	1	0.625
	JSU	*He>Heq*	3	3	3	2
		*Sign test*	0.657	0.66	0.674	0.332
		*Wilcoxon test*	1	0.813	0.813	0.219
	SIC	*He>Heq*	2	2	1	0
		*Sign test*	0.323	0.325	0.99	0.013*
		*Wilcoxon test*	0.625	0.219	0.094	0.031*
	SHX	*He>Heq*	3	3	3	2
		*Sign test*	0.647	0.677	0.659	0.332
		*Wilcoxon test*	0.813	0.813	1	0.219

TMP, two-phase mutation model with (indels larger than one repeat of 30%, 20% and 10%, respectively);

a , single step mutation; SMM, stepwise mutation model. *He>Heq*, number of loci showing a heterozygote excess (out of 5 loci tested in each samples).

*,*P*<0.05 (two tails P-value for deviation from MDE).

Estimates of long-term *Ne* varied considerably depending on the model used. Under the heterozygote excess model all of the *Ne* estimates were infinity. Under the linkage disequilibrium model, diverse *Ne* values were detected across populations (Table 7). The two clusters showed similar variability of *Ne,* from ∞ (CHQ) to 18.4 (SHD) in the cluster I and ∞ (HUB) to 14.2 (LIA) in the cluster II, respectively.

**Table 7:** Estimated *Ne* based on the linkage disequilibrium model.

Cluster	Population	*Ne*	95%CI
I	HEN	48.4	25.3–210.9
	HUB	∞	166.9-∞
	YUN	250.4	73.9-∞
	GUD	117.8	48.5-∞
	SHD	18.4	12.3–31.7
	GZH	60.6	32.8–226.7
II	CHQ	∞	61.3-∞
	FUJ	105.8	35.6-∞
	JSU	27.7	18.2–50.3
	LIA	14.3	7.9–41.3
	GXI	35.3	16.8–446.8
	SHX	29.3	17.8–65.7
	SIC	63.4	29.7–2739.5
	HAN	91.6	28.2-∞

CI, confidence intervals; ∞, infinity.

## Discussion

Sampling strategy and geographic coverage greatly influence the analysis and interpretation of the data generated from the samples. *A. sinensis* occur in most parts of China, a range as from 100° E to 120° E, and from 19° N to 54° N [22]. In this study, *A. sinensis* mosquitoes were collected from most localities across its range in China. The LIA was at the most northern limit, and HAN was at the most southern limit of the distribution.

The five microsatellites in *A. sinensis* [30] are highly polymorphic in the populations, and thus are useful for exploring *A. sinensis* population genetic structure. Theses microsatellite loci have not been physically mapped to *A. sinensis* polygene chromosomes. Therefore, their location with respect to polymorphic chromosome forms is unknown, but linkage disequilibrium between loci was detected only in 3.6% of 140 comparisons suggests that they are at least statistically independent and might distribute across the genome. The high allelic diversity and expected heterozygosity were observed in most of populations, which was similar to the level of diversity in the *A. darlingi* in Peru and Brazil (*H_O_*  = 0.742, 0.457) [24], *A. albimanus* in Latin America (*H_O_*  = 0.73) [33], African vectors *A. gambiae* (*H_O_*  = 0.59) [34] and *A. funestus* (*H_O_*  = 0.672, 0.529) [35], [36]. In China, *A. sinensis* occurs in the temperate climate zones, populations undergo marked seasonal variations in abundance, reaching high densities only during the summer months. The high level of genetic diversity suggests that *A. sinensis* are able to maintain a relatively large effective population size in spite of the seasonality of low temperature in cold winter.

In this study, significant deviations from HWE due to the heterozygote deficits were detected in most samples. These could be attributed to the Wahlund effect, inbreeding, selection or null alleles. Selection was not considered because it usually occurs in one locus, not systemically in multiple loci as detected in this study. Inbreeding has genome-wide effect. If inbreeding were important in our case, we would expect a similar heterozygosity deficiencies in all markers with each population studied, which we did not detect. The reduction of heterozygotes at several loci detected in this study is likely the results of presence of null alleles that are not amplified because of the mutations at the primer annealing sites. In this study, there were specimens that failed to amplify some alleles at certain loci, but efficiently amplified at other loci. The Wahlund effect, referring to subpopulations in a sample, is likely another cause for the heterozygote deficits. Indeed, the Bayesian analysis revealed two gene pools across the locations sampled (Figure 2), and the two gene pools coexist in at least six collections (Table 3). Thus, a part of the heterozygote deficits detected in these samples could be the result of the Wahlund effect.

The two genetic divisions of *A. sinensis* were represented by two population clusters. There is substantial differentiation between two gene pools, demonstrated by mean *F*_ST_ *= *0.156 between the two clusters (Table 4) and pairwise comparisons (*F*_ST_ *= *0.120–0.201) among populations between the two clusters (Table 5). The gene flow was remarkably limited between the clusters (*Nm*  = 1–1.7). The level of differentiation is comparable to that detected previously between *A. sinensis* populations in China using isozymes and RAPD markers (*F*_ST_  = 0.069–0.111) [13], [28], between *A. albimanus* populations from Central and South America (*F*_ST_ = 0.114) [33], among *A. gambiae* populations in west Africa separated by >200 km (*F*_ST_  = 0.034–0.167) [37], and among *A. darlingi* populations from Brazil and Peru [24] as well as between *A. funestus* populations from west, central, and eastern Africa (*F*_ST_  = 0.110) [7]. The distribution of two clusters appears no noticeable geographic patterns, and no correlation between genetic and geographic distance was detected by the Mantel test (Figure 3). In addition, the sympatric occurrence of two gene pools was found in 6 of 14 collections (Table 3). Therefore, the differentiation between the two population clusters probably was not influenced by geographic distance and barriers (*e.g.*, Yangtze River, Yellow River and Qinling Mountains).

Within the population clusters, pairwise differentiation was little or low (*F*_ST_ = 0.004–0.048). No isolation by distance was detected (Figure 3). However, a large amount of variability in *Ne* among the populations (14.4-∞ in the cluster I and 18.4-∞ in the cluster II, Table 7) suggests that ecological and/or historical heterogeneity may contribute to the differentiation observed among these populations. Similarly, *Ne* heterogeneity has been reported to be associated with the population differentiation for *A. darling* [24] and *A. albimanus* [38]. In the cluster II, there is some evidence of a population expansion in SIC and GXI (Table 7). Taken together, the population diversity in the form of two clusters may be caused largely by the differences in *Ne* among the populations and/or different demographic [8].

This study revealed that *A. sinensis* populations across China are primarily structured with two major genetic units. The distribution pattern could not be attributed to the isolation by distance. The wide range and sympatric distribution of the two genetic pools suggest that these two gene pools may have been segregating in the *A. sinensis* populations for a relative long time. Our findings emphasize the need for further investigation with deeper sampling (especially in the areas both gene pools exist) and more genetic loci in order to thoroughly elucidate the forces that shape and maintain the population structure. More studies are required to characterize the two gene pools of *A. sinensis* regarding ecology and malaria susceptibility. The population structure of *A. sinensis* implies that the expansion and spread of genes responsible for immunity against malaria or insecticide resistance would be easier within than between the genetic units. Moreover, the estimate of effective population size would help to evaluate the effectiveness of mosquito control measurements [8].

## Materials and Methods

### Mosquito collections and species identification

Wild adult *A. sinensis* were collected from 1996 to 2008, by using indoor light traps at livestock corrals. Human landing catches at human living room were tried in 1996 in Sichuan (SIC) and Hainan (HAN). Only two specimens were caught by human bait in SIC. *A. sinensis* is zoophilic, therefore, the human bait was no longer used in later collections. The 20 collection sites in 14 provinces in China were located from 109°58′ N to 120°25′ N, and 23°60′ E to 31°44′ E (Table 1, Figure 1). Five samples, YUN, HUB, LIA, SHD and SIC, consisted of specimens pooled from two or three sites in proximity to each other, as stated in Table 1. The distances between sites were 25–100 km.

Mosquitoes of *A. hyrcanus* group were identified by morphology using the identification keys of Lu *et al.* [22]. Specimens were kept individually in silica gel filled tubes at 4°C, until DNA extraction was performed according to Collins *et al.* [39]. *A. sinensis* species identification was done by a PCR assay based on ribosomal DNA ITS2 markers previously described in Ma *et al.* [32].

### Genotyping and data analysis

Five microsatellite loci, ANS1, ANS5, ANS6, ANS11 and ANS15 [30], were used for genotyping. Each locus was amplified by PCR using fluorescently labeled (FAM, NED, or HEX) forward primers. Amplified fragments were separated by capillary electrophoresis in an automatic sequencer (ABI 3770, Applied Biosystems, Foster City, CA) and size scored using GENOTYPER 3.7 software (Applied Biosystems, Foster City, CA).

Genetic diversity within samples and overall was measured at each locus by estimates of allele frequencies, number of alleles *A,* allele richness *Rs*, inbreeding coefficient *F*_IS_, expected heterozygosity *H*_E_, and observed heterozygosity *H*o [40], using the software FSTAT 2.9.3.2 [41]. Within each locality the frequency of null alleles was determined using the Brookfield 2 estimate [42], and the allele and genotype frequencies were then adjusted accordingly in MICRO-CHECKER 2.2.3 [43]. The null allele-adjusted dataset was compared to the original dataset to investigate the impact of null alleles on estimations of genetic differentiation. Genotypic frequencies were tested against Hardy-Weinberg equilibrium (HWE) for each locus in the pooled population and in each sample. Statistical significance was assessed by the exact probability test available in GENEPOP 3.2 [44]. Linkage disequilibrium between loci was tested by exact tests on contingency tables, also available in GENEPOP.

Genetic differentiation was estimated by calculating Wright's *F* statistics (*F*_ST_, *F*_IS_, *F*_IT_) values per locus between clusters and pairs of populations using ARLEQUIN 2.001 [45] and GENEPOP. The number of migrants per population per generation (*Nm*) between localities was estimated from pairwise *F*_ST_ [46]. An analysis of molecular variance (AMOVA) was used to examine the distribution of genetic variation in Arlequin using *F*_ST_. We focused on estimates of *F*_ST_ performed under the infinite alleles model (IAM) because this model is considered more reliable when fewer than 20 microsatellites are used [47]. The significance for all calculations was assessed by 10,000 permutations and the *P*-values. The isolation by distance model was investigated as a potential explanation for the observed population differentiation. The significance of the regression of genetic differences on geographic distance between sample pairs was tested using a Mantel test [48] with 100,000 permutations using GENEPOP.

A Bayesian approach was used to infer the number of clusters (*K*) in the data set without prior information of the sampling locations, implemented with STRUCTURE 2.2 [49]. A model where the allele frequencies were correlated within populations was assumed (λ was set at 1, the default value). The software was run with the option of admixture, allowing for some mixed ancestry within individuals, and α was allowed to vary. Twenty independent runs were done for each value of *K* (*K* = 1 to 9), with a burn-in period of 100,000 iterations and 100,000 replications. The method of Evanno *et al.* [50] was used to determine the most likely number of clusters. This approach uses an *ad hoc* quantity, Δ*K*, based on the second order rate of change of the likelihood function between successive values of *K*.

Because demographic instability such as recent population bottleneck and/or expansion might bias genetic differentiation estimates to a significant extent [51], [52], heterozygosity tests [53] were used to detect deviations from mutation-drift equilibrium (MDE). These tests compare two estimates of expected heterozygosity, one based on allele frequencies (*He*), assuming Hardy-Weinberg proportions, and another based on the number of alleles and sample size (*Heq*), assuming MDE. At MDE, both estimates should be similar in the majority of loci analyzed (*i.e. He = Heq*). If a population experiences a bottleneck, rare alleles will be rapidly lost and therefore *Heq* will decrease faster than *He* (*i.e. He* > *Heq*). This apparent excess of heterozygosity in a significant number of loci is an indicator of a bottleneck, whereas the converse (*i.e. He* <*Heq*) may indicate a population expansion. Estimates of expected heterozygosity under MDE were calculated under the Stepwise Mutation Model (SMM) and Two Phase Models (TPM) with 10%–30% indels larger than the repeat unit. Calculations were done using the software BOTTLENECK 1.2.02. [53]. The long-term effective population size (*Ne*) was estimated using NEESTIMATOR 1.3 [54] based on the heterozygote excess and linkage disequilibrium models.

## Supporting Information

Figure S1 **Allele distributions across populations for each of five loci.** Alleles are denoted by length (bp), and populations are color coded.
(TIF)

Figure S2 **Differentially distributed alleles across populations between two clusters.** Top, ANS1; Middle, ANS5; Bottom, ANS6. Differential alleles are color coded, and populations were arranged based on their cluster assignment.
(TIF)
