# Supplementary figures and images for: Population Structure of the Malaria Vector Anopheles sinensis (Diptera: Culicidae) in China: Two Gene Pools Inferred by Microsatellites
Source: PLoS One. 2011 Jul 22;6(7):e22219. doi: 10.1371/journal.pone.0022219 (PMC3142120; doi:10.1371/journal.pone.0022219)

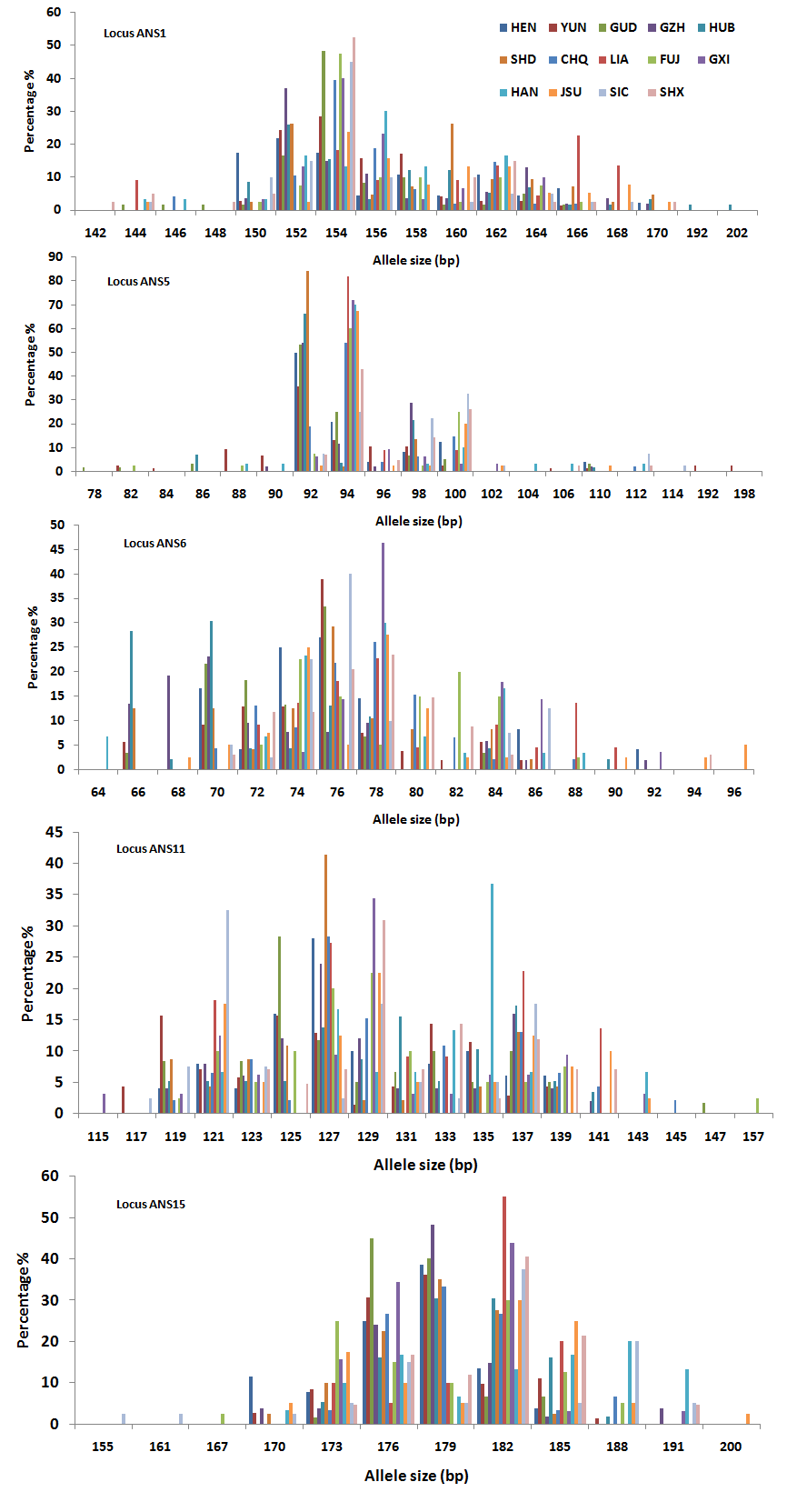

Supplement: Figure S1 — Allele distributions across populations for each of five loci. Alleles are denoted by length (bp), and populations are color coded. (TIF) [file pone.0022219.s001.tif]

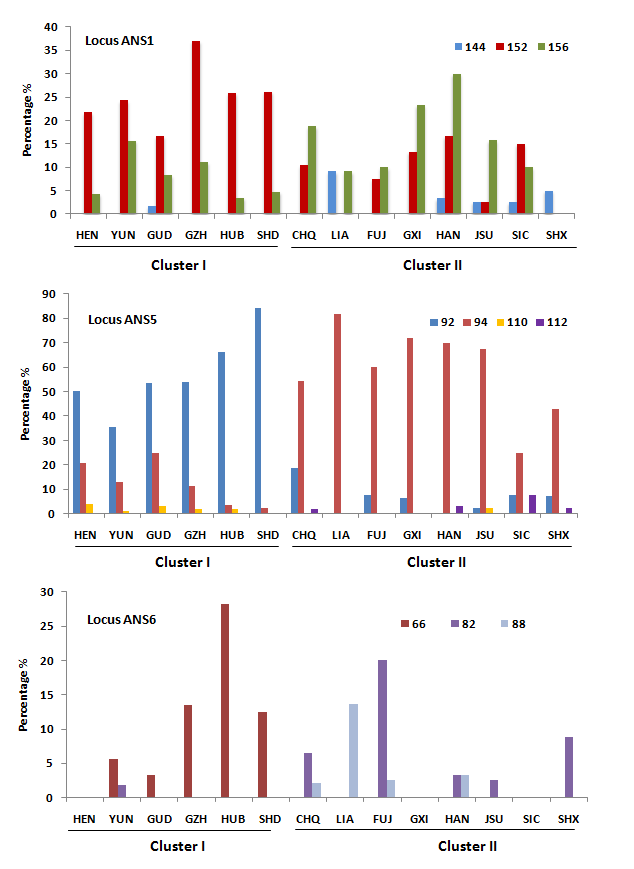

Supplement: Figure S2 — Differentially distributed alleles across populations between two clusters. Top, ANS1; Middle, ANS5; Bottom, ANS6. Differential alleles are color coded, and populations were arranged based on their cluster assignment. (TIF) [file pone.0022219.s002.tif]
